# Genetic Relationships Between Ethanol-Induced Conditioned Place Aversion and Other Ethanol Phenotypes in 15 Inbred Mouse Strains

**DOI:** 10.3390/brainsci9080209

**Published:** 2019-08-20

**Authors:** Christopher L. Cunningham

**Affiliations:** Department of Behavioral Neuroscience and Portland Alcohol Research Center, Oregon Health & Science University, Portland, OR 97239, USA; cunningh@ohsu.edu

**Keywords:** alcohol, reinforcement, reward, place aversion, taste aversion, drinking, withdrawal, locomotor activity, learning, inbred strains

## Abstract

The genetic relationships between different behaviors used to index the aversive effects of ethanol are unknown. To address this issue, ethanol-induced conditioned place aversion (CPA) was tested in a genetically diverse panel of 15 inbred mouse strains. Mice were exposed to an unbiased place conditioning procedure using ethanol doses of 0, 2, or 4 g/kg; all injections were given immediately after 5-min exposure to distinctive tactile cues. There were dose-dependent effects of ethanol on CPA and on the change in pre-injection activity rates between the first and last conditioning trials. Most strains (80%) developed CPA, demonstrating the generalizability of this behavior. Moreover, genotype had significant effects on CPA magnitude and locomotor activity rates. Strain means from this study and previously published studies were then used to examine genetic correlations. These analyses showed significant genetic correlations between CPA and ethanol intake/preference, conditioned taste aversion, and drug withdrawal (but not blood ethanol concentration or conditioned place preference), supporting the idea of commonality in the genes underlying CPA and each of these behaviors. The overall pattern of findings is consistent with previous data suggesting that genetic differences in sensitivity to ethanol’s aversive effects play a role in determining strain differences in ethanol drinking. The broader implication is that individuals who are more sensitive to the aversive effects of ethanol may be protected from developing the excessive drinking behaviors characteristic of alcohol use disorders.

## 1. Introduction

Genotype affects a wide array of responses to ethanol in animals, including changes in locomotor activity, body temperature, ataxia, and loss of righting reflex [1,2]. Given the well-established influence of genotype on alcoholism in humans [3], attention in animal models has focused particularly on genetic variation in phenotypes thought to be relevant for understanding the development, maintenance, and relapse of excessive drinking. These phenotypes have included such things as ethanol self-administration in operant procedures and ethanol drinking (e.g., [4,5,6,7]), ethanol withdrawal severity [8,9,10], as well as ethanol’s rewarding and aversive effects as indexed by conditioned place preference (CPP) and conditioned taste aversion (CTA) procedures [11,12,13,14]. Overall, these studies have revealed substantial genetic differences in sensitivity to alcohol’s rewarding and aversive effects, thereby offering support for the suggestion that vulnerability to alcohol addiction depends, at least in part, on the biological mechanisms that underlie genetic variation in these phenotypes.

Historically, substantial theoretical weight has been given to the role played by the positive motivational (rewarding, reinforcing) effects of ethanol in the development of alcohol use disorder (e.g., [15]). However, ethanol is also well known to produce negative motivational (aversive, punishing) effects that could bestow a protective effect that reduces the likelihood of developing an excessive drinking pattern [16]. The latter possibility is well supported by rodent studies that show a negative genetic relationship between ethanol consumption or preference and sensitivity to ethanol-induced conditioned taste aversion (CTA) [13,17,18]. That is, mouse genotypes that are more sensitive to ethanol-induced CTA tend to drink less ethanol compared to mouse genotypes that are less sensitive to ethanol-induced CTA.

To date, CTA is the only measure of ethanol’s aversive effect that has been examined for its genetic relationship to other ethanol-related phenotypes. The present study was designed to remedy this situation by extending the generalizability of findings from the CTA studies to a different measure of ethanol aversion that does not involve ingestive behavior. More specifically, this study examined mouse strain differences in development of conditioned place aversion (CPA) to tactile cues paired with post-trial injection of ethanol (0, 2, or 4 g/kg) in an unbiased Pavlovian discrimination conditioning procedure. This procedure is based on several previous studies showing that injection of ethanol immediately after brief (5-min) exposure to a distinctive tactile floor cue will establish an aversion to that cue in DBA/2J mice, relative to an alternative cue that has been paired with vehicle (saline) injection [19,20,21,22,23,24].

Two different approaches have been used in the past to study genetic correlations between different drug-induced behavioral phenotypes [25]. One approach involves testing lines of rats or mice that have been selectively bred for high or low sensitivity to a specific drug effect. Because successful selective breeding is assumed to homozygously fix genes that influence the selection phenotype while not affecting phenotype-irrelevant genes, any differences between the selected lines on some other phenotype are thought to reflect the action of common genes. In other words, the two phenotypes are genetically correlated. The second approach for estimating genetic correlations involves correlating strain means for different phenotypes measured across a large panel of inbred strains. Inbred strains are created by brother–sister mating for at least 20 generations, which results in strains of genetically identical individuals. Strain means are used for estimating genetic correlations because strain mean differences are primarily attributable to differences in genotype when strains are tested under common environmental conditions. When using inbred strain means to estimate genetic correlations, it is important to include a relatively large number of strains to reduce the influence of unique phenotype-irrelevant genes that may have become randomly fixed in the creation of each inbred strain.

In order to reflect a broad range of mouse genotypes, and to increase power for detecting genetic correlations with ethanol-related phenotypes measured in previous studies, a genetically diverse panel of 15 inbred strains was tested here. The same 15 strains were used in previous studies of ethanol-induced CTA [13] and ethanol-induced CPP [14]. It was predicted that ethanol-induced CPA would show a positive genetic correlation with CTA and a negative genetic correlation with ethanol consumption, but that there would be no genetic correlation with ethanol-induced CPP.

## 2. Materials and Methods

### 2.1. Subjects

The subjects were adult male mice from 15 standard inbred strains: 129P3/J (formerly 129/J), A/HeJ, AKR/J, BALB/cJ, C3H/HeJ, C57BL/6J, C57L/J, C58/J, CBA/J, DBA/1J, DBA/2J, NZB/B1NJ, PL/J, SJL/J, and SWR/J. These strains were chosen to maximize the number of strains overlapping with those used in other multi-strain studies of ethanol-related phenotypes [5,10,13,14]. The mice were obtained from the Jackson Laboratory (Bar Harbor, ME) at 4–6 weeks of age and were allowed to acclimate to the colony for 2–4 weeks before conditioning. Subjects were housed in same-strain groups of two to five in polycarbonate cages (27.9 × 9.5 × 12.7 cm) with cob bedding. Rodent chow and water were freely available in the home cages and ambient temperature was maintained at 21 ± 1 °C. All experimental procedures were conducted during the light phase of a 12:12-hour light:dark cycle (lights on at 07:00).

A total of 923 mice received conditioning trials in this experiment. Mice were approximately 56 days old at the start of testing. A total of 36 subjects (3.9%) were excluded from the final data analysis for various reasons: (a) Procedural error (number of mice (*n*) = 1); (b) ill health or death, most likely caused by injection injury or cage mate attacks (*n* = 26); or (c) vendor error or low availability (*n* = 9). Subject attrition was distributed across 11 of the 15 strains, with the largest attrition in the A/HeJ strain, primarily due to a vendor error and the elimination of the 2 g/kg dose group due to limited availability (see Appendix A). The final number of mice in each strain that completed testing ranged from 59 to 63, except for A/HeJ (*n* = 27). The same experimenter tested all of these mice across a 9-month period in 11 cohorts that ranged in size from 53 to 96. Each cohort contained mice from 5 to 12 different strains and each strain was represented in at least four different cohorts. Given the many different coat colors across this set of strains, it was not possible to blind the experimenter to strain. Experimental procedures were conducted in accordance with the National Institutes of Health Principles of Laboratory Animal Care, and the OHSU Institutional Animal Use and Care Committee approved the protocol.

### 2.2. Apparatus

The apparatus has been described in detail elsewhere [26]. Briefly, it consisted of 12 identical acrylic and aluminum boxes (30 × 15 × 15 cm) enclosed in separate light- and sound-attenuating enclosures (Coulbourn Instruments Model E10-20). Six sets of infrared light sources and detectors were mounted opposite each other at 5-cm intervals on the long walls of each box, 2.2 cm above the floor. A microcomputer recorded activity and position (left vs. right).

The floor of each box consisted of interchangeable halves made of one of two textures. The “grid” (G) floor was composed of 2.3 mm stainless steel rods mounted 6.4 mm apart in acrylic rails. The “hole” (H) floor was made from perforated stainless steel (16 GA) with 6.4-mm round holes on 9.5-mm staggered centers. These floor textures were selected on the basis of previous studies showing that drug-naive groups of C57BL/6J and DBA/2J mice spend about half their time on each floor type during preference tests [11,27]. The apparatus and floors were wiped down with a damp sponge and the litter paper was changed after each animal.

### 2.3. Procedure

There were three phases in this experiment: Habituation (one session), conditioning (eight sessions), and testing (one session). Sessions were conducted 5 days per week with a 2-day break between the first four and second four conditioning sessions.

#### 2.3.1. Habituation

The habituation session was intended to reduce the novelty and stress associated with handling and exposure to the apparatus. All mice were weighed and immediately placed in the center of the conditioning box on a smooth floor covered with paper. After a 5-min exposure to the apparatus and paper floor, mice were removed and returned to the home cage. Subjects were not exposed to the distinctive floor textures during the habituation session in an attempt to avoid latent inhibition.

#### 2.3.2. Conditioning

During the conditioning phase, mice from each of the 15 strains were randomly assigned to one of two ethanol dose groups (2 or 4 g/kg) or to a saline control group. As noted earlier, however, A/HeJ mice were assigned only to the saline and 4 g/kg groups. Dose was manipulated by varying the volume (12.5 or 25.0 ml/kg) of a 20% v/v solution of ethanol in saline [28]. These doses were chosen on the basis of several previous studies indicating that they produce a reliable conditioned aversion in DBA/2J mice under training conditions similar to those used here [19,20,21,22,23,24]. Additionally, these doses matched those used in 15-strain studies that examined ethanol-induced CPP [14] and CTA [13]. Saline only groups (0 g/kg) received saline injections in the same volume (25 ml/kg) as the high dose groups. All injections were intraperitoneal.

Within each dose group, mice were randomly assigned to one of two conditioning subgroups and exposed to an unbiased place conditioning procedure [29]. Mice had access to the whole apparatus and floor texture was identical on both sides during all conditioning trials (i.e., one-compartment procedure). Each mouse was placed in the center of the apparatus for each conditioning session. On alternate days, mice in the G+ conditioning subgroups were weighed and injected with ethanol after removal from the grid floor (CS+ trial), and with saline after removal from the hole floor (CS–trial). These contingencies were reversed for mice in the G–subgroups. The saline control group was injected with saline after all trials, regardless of the alternating floor type. Four 5-min conditioning trials of each type were given over an 8-day period. The order of exposure to CS+ and CS−was counterbalanced within each conditioning subgroup. Because the conditioning subgroups within each strain were matched for overall exposure to each floor type, and differed only in the floor–drug contingency, any differences between subgroups during preference tests can be attributed to learning based on the Pavlovian relationship between the CS+ floor and ethanol [30].

#### 2.3.3. Place preference tests

A 30-min floor preference test was given 24 h after the last conditioning trial. The apparatus was configured with half grid floor and half hole floor. Floor position was counterbalanced within each conditioning subgroup. All mice were removed from the home cage and placed immediately in the apparatus without weighing or injection. Total time spent on the grid floor was the primary dependent variable.

#### 2.3.4. Data Analyses

Data were evaluated using analysis of variance (ANOVA). Strain, Dose and Conditioning Subgroup (G+ vs. G−) were treated as between-group factors; Trial and Trial Type were included as repeated measures. All 15 strains were included in overall analyses, except for those involving the 2-g/kg groups, which did not include A/HeJ mice for reasons noted earlier. The coefficient of genetic determination (i.e., the sum of squares between strains divided by the total sum of squares from a one-way ANOVA) was used to estimate heritability (*h*) for each phenotype [5]. Genetic (Pearson) correlations were calculated using the phenotype means for each strain [25]. For example, to estimate the genetic correlation between CPA and locomotor activity, the 15 strain means for CPA (x-score) were paired with the corresponding 15 strain means for activity (y-score) and a Pearson correlation coefficient (r) was computed. Differences among individuals within-strain provided a measure of non-genetically determined variation in behavior.

## 3. Results

### 3.1. Habituation Session Activity

Mean activity rates for each strain during the habituation session are listed for the 0-, 2-, and 4-g/kg groups in Supplementary Tables S1–S3, respectively. Habituation session activity differed widely by strain, with overall strain means (collapsed across dose) ranging from 32 to 129 activity counts/min. One-way ANOVA indicated a significant difference among strains [F(14, 864) = 139.7, *p* < 0.001]. As shown in the tables, heritability of the habituation session activity phenotype was relatively high in each dose group (0.66–0.74).

### 3.2. Conditioning Trial Activity

**Figure 1:**
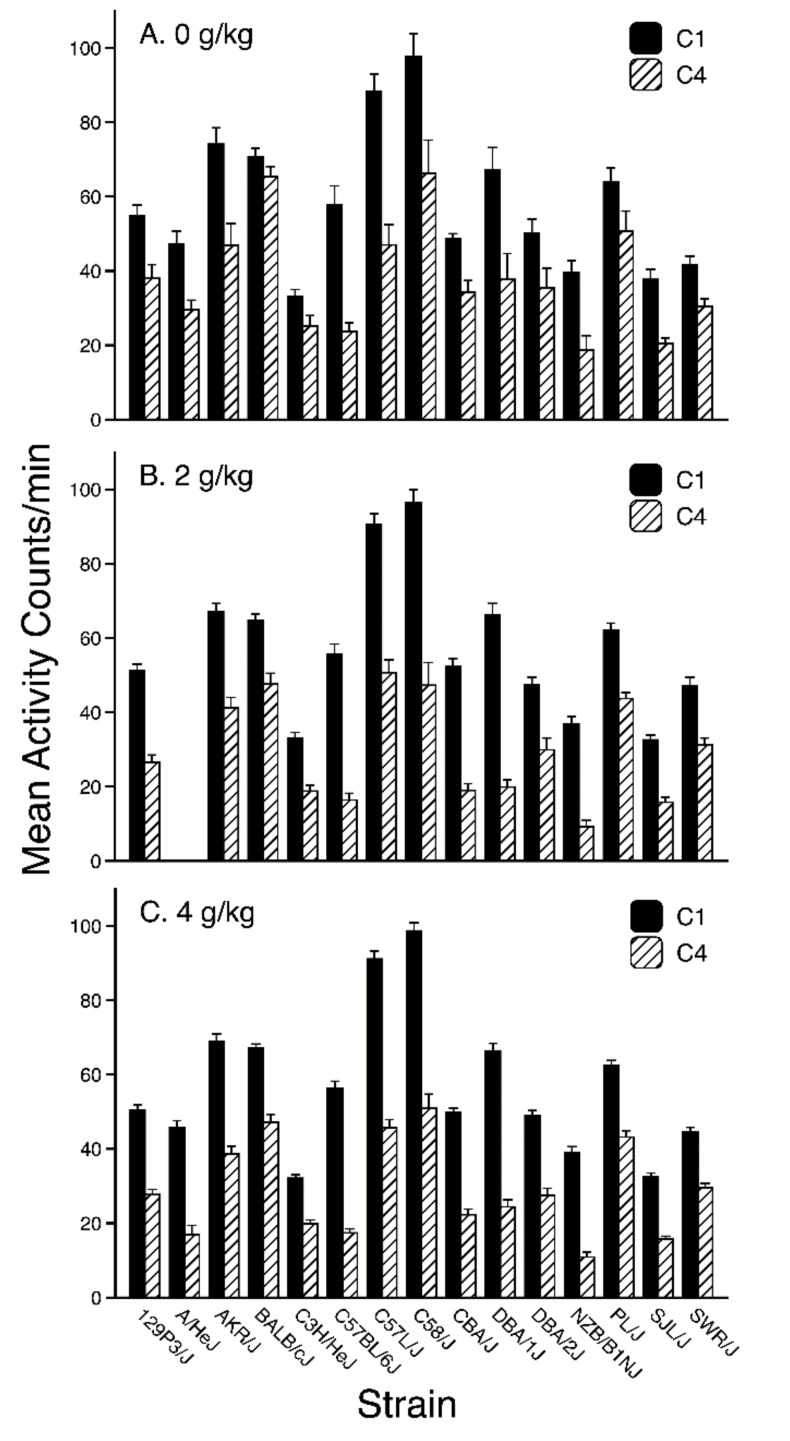
Mean (+ SEM) activity rates (counts per minute) on the first (C1: Solid bars) and last (C4: Cross-hatched bars) pairs of conditioning trials for each strain within the (**A**) 0 g/kg, (**B**) 2 g/kg, and (**C**) 4 g/kg dose groups (collapsed across conditioning subgroups). Activity was averaged across the CS+ and CS−trials since there was no effect of trial type. Each bar for the 0-g/kg group represents 9–13 mice, whereas each bar for the ethanol-injected groups represents 18–25 mice.

**Figure 2:**
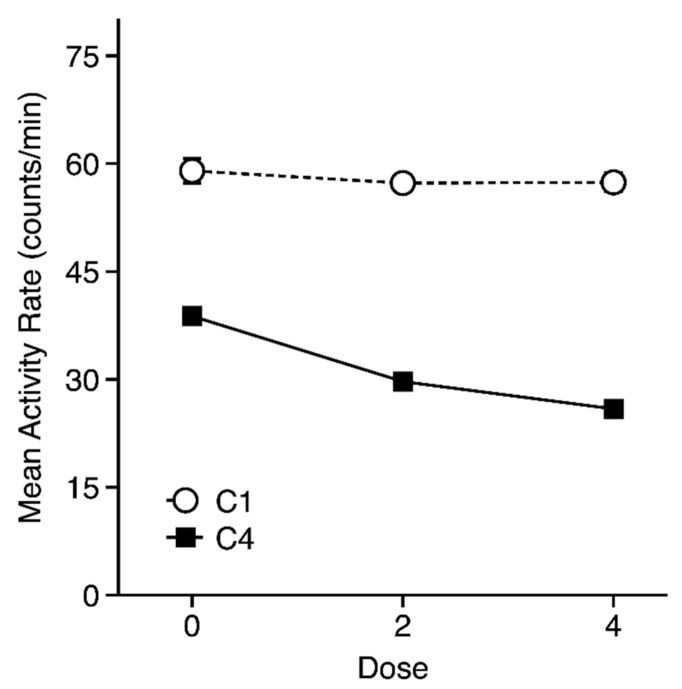
Mean activity rates (±SEM) in each dose group during the first (C1: Open symbols) and last (C4: Closed symbols) conditioning trials. Data are collapsed over strain, conditioning subgroup, and trial type. Each point for the 0-g/kg group includes 171 mice, while each point for the ethanol-injected groups includes 338–343 mice.

A preliminary four-way repeated measures ANOVA (Strain × Dose × Trial Type × Trial) was applied to activity data from the first and last conditioning trials (omitting the A/HeJ strain). This preliminary analysis showed no main effect or interactions involving the Trial Type factor, indicating that activity rates were similar on trials that immediately preceded saline (CS−) or ethanol (CS+) injections (see Supplementary Tables S2 and S3 for CS− and CS+ means for each strain). Thus, to simplify presentation and analysis of the conditioning trial data, a single activity rate was calculated for each mouse at each point during conditioning by averaging the rates across the CS+ and CS−trials. Strain means (±SEM) for these activity rates are depicted for each Strain × Dose group in Figure 1. As can be seen, all strains showed a decrease in activity between the first and last conditioning trial. Moreover, that decrease was generally greater in the two groups that received ethanol injections on CS+ trials (see Figure 2), although not in all strains. These observations were supported by three-way repeated measures ANOVA (Strain × Dose × Trial) that yielded significant main effects of all three factors [Strain: F(13, 810) = 122.5, *p* < 0.001; Dose: F(2, 810) = 26.0, *p* < 0.001; Trial: F(1, 810) = 2389.8, *p* < 0.01]. All interactions involving the Trial factor were also significant [Strain × Trial: F(13, 810) = 31.1, *p* < 0.001; Dose × Trial: F(2, 810) = 31.4, *p* < 0.01; Strain × Dose × Trial: F(26, 810) = 1.9, *p* < 0.01], but the Strain × Dose interaction was not significant (*p* > 0.40).

Separate two-way ANOVAs (Strain × Dose) were conducted on the data for each trial to help interpret the three-way interaction. Analysis of the first trial showed only a significant main effect of Strain [F(13, 810) = 139.7, *p* < 0.001], while analysis of the last trial revealed a significant Strain × Dose interaction [F(26, 810) = 1.73, *p* < 0.02] in addition to significant main effects of Strain [F(13, 810) = 51.5, *p* < 0.001] and Dose [F(2, 810) = 50.0, *p* < 0.001]. Post-hoc testing of the Dose factor on the fourth trial indicated that each dose group differed significantly from both of the other dose groups (all Bonferroni-corrected *p*’s < 0.001). Simple effect tests of the Dose factor for each Strain on the fourth trial showed a significant dose effect in the following strains (all *p*’s < 0.05): 129P3/J, AKR/J, BALB/cJ, C57L/J, C58/J, CBA/J, DBA/1J, DBA/2J, and PL/J.

### 3.3. Place Aversion Test

The test data were analyzed using two different ways of characterizing place conditioning performance [26,29,31]. First, raw time scores (i.e., s/min on grid floor) were analyzed using Strain, Dose, and Conditioning Subgroup as factors. The conditioning subgroups were matched for overall exposure to the floor cues, handling, and injection. Thus, significant differences between subgroups can be attributed to the paired relationship between ethanol and the CS+ floor, allowing us to determine whether place conditioning occurred within each strain [26,29,31]. In addition, to simplify strain and dose comparisons, we examined percent time scores [(s/min on ethanol-paired floor ÷ 60) × 100] using Strain and Dose as factors collapsed across conditioning subgroup. Data from the A/HeJ strain were excluded from overall ANOVAs that included Dose as a factor, but were included in follow-up analyses that did not involve the 2-g/kg groups.

Strain means for time spent on the grid floor are listed for the saline (0 g/kg) group from each strain in Supplementary Table S1. Overall, the saline-only groups spent an average of 30.2 ± 0.6 s/min (50.3%) on the grid floor during the preference test. Since saline-only mice were exposed to each floor type as often as ethanol-treated mice, their performance reflects unconditioned preference for the two floor types in the absence of ethanol exposure. As can be seen, most strains (14/15) had preference scores in the middle of the response range (30 ± 4.0 s/min on grid), but one strain showed a stronger bias in favor of the hole floor (NZB/B1NJ). One-way ANOVA indicates a significant difference in unconditioned floor preference among strains [F(14, 167) = 2.4, *p* < 0.01].

**Figure 3:**
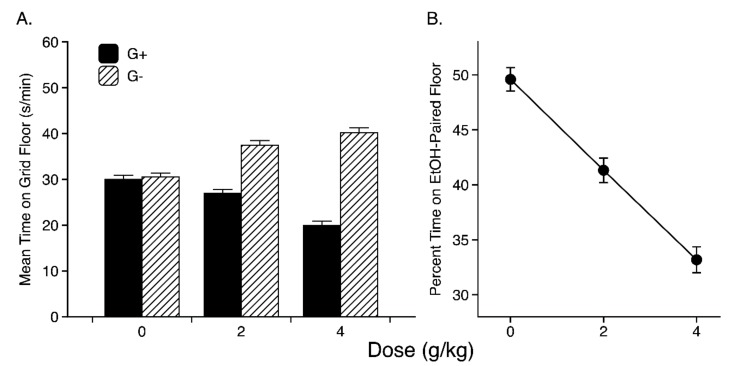
Conditioned Place Aversion Test: (**A**) Mean s/min (+SEM) spent on the grid floor by the G+ and G− conditioning subgroups at each ethanol dose (collapsed across strain). Each bar depicts 90–92 mice (0 g/kg group) or 171–181 mice (2 and 4 g/kg groups). (**B**) Mean percent time (±SEM) spent on the ethanol-paired floor (collapsed across conditioning subgroup) at each ethanol dose (collapsed across strain). Each point depicts 182 mice (0 g/kg group) or 347–358 mice (2 and 4 g/kg groups).

Strain means for the conditioning subgroups given 2- or 4-g/kg ethanol injections are listed in Supplementary Tables S4 and S5, respectively. Overall, ethanol-treated mice in the G+ subgroups spent less time on the grid floor than ethanol-treated mice in the G− subgroups, indicating that both ethanol doses induced a conditioned floor aversion [19]. The effect of ethanol dose on time spent on the grid floor is depicted in the left panel of Figure 3, which shows that the difference between the two conditioning subgroups increased as ethanol dose was increased. Three-way ANOVA (Strain × Dose × Conditioning Subgroup) applied to the time scores yielded significant main effects of each factor [Strain: F(13, 776) = 3.3, *p* < 0.001; Dose: F(2, 776) = 3.9, *p* < 0.05; Conditioning Subgroup: F(1, 776) = 160.1, *p* < 0.001], as well as significant Strain × Conditioning Subgroup [F(13, 776) = 3.4, *p* < 0.001] and Dose × Conditioning Subgroup [F(2, 776) = 44.7, *p* < 0.01] interactions. The three-way interaction, however, fell short of the criterion for significance [F(26, 776) = 1.5, 0.05 < *p* < 0.06]. Results of planned comparisons between the two conditioning subgroups within each strain are shown for the ethanol-treated groups in Supplementary Tables S4 and S5. These analyses indicate significant CPA in 8 strains (57%) at the 2 g/kg dose and in 12 strains (80%) at the 4 g/kg dose.

**Figure 4:**
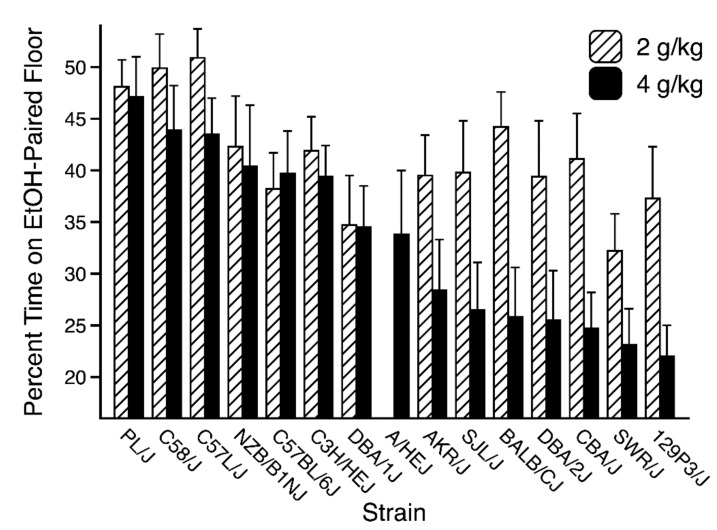
Conditioned Place Aversion Test. Mean percent time (+SEM) spent on the ethanol-paired floor (collapsed across conditioning subgroup) by each strain at each ethanol dose. Each bar depicts 9–13 mice (0 g/kg groups) or 18–26 mice (2 and 4 g/kg groups).

As noted above, raw time data for each mouse in both conditioning subgroups were also converted to percent time on the ethanol-paired floor and averaged to create a single preference score for each strain at each dose. The preference scores calculated for the saline (0 g/kg) group were arithmetically identical to those computed for the ethanol-treated groups, but the raw time used for the numerator was determined randomly in a balanced manner (i.e., time on the grid floor was randomly used as the numerator for half of the mice in each strain, while time on the hole floor was used for the other half). To better illustrate the continuous nature of the conditioned aversion phenotype, strains were ordered by the magnitude of CPA measured in the 4-g/kg groups in Figure 4. As can be seen, some strains showed little or no CPA at either dose (e.g., PL/J, C58/J, and C57L/J), while other strains showed a substantial aversion, spending about 25% of the test session on the floor that was previously paired with post-trial injection of ethanol (e.g., DBA/2J, CBA/J, SWR/J, 129P3/J). No strain showed development of a conditioned preference for the ethanol-paired floor. Moreover, CPA magnitude was dose-dependent, with stronger aversion induced by the higher dose, although several strains showed little or no difference between doses. In no case did 4 g/kg produce an appreciably weaker aversion than that seen at 2 g/kg in the same strain.

Two-way (Strain × Dose) ANOVA of the percent time data revealed significant main effects of Strain [F(13, 818) = 3.3, *p* < 0.001] and Dose [F(2, 818) = 43.0, *p* < 0.001], but no significant Strain × Dose interaction [F(26, 818) = 1.4, *p* > 0.10], indicating that the Dose effect was statistically similar across strains. Post-hoc pairwise comparisons showed that each dose group differed significantly from both of the other dose groups (all Bonferroni-corrected *p*’s < 0.001). Ethanol’s systematic dose-related effect on CPA is well illustrated in the right-hand panel of Figure 3, which shows mean test preference at each dose, collapsed across strain and conditioning subgroup.

As noted above, comparison of raw time scores between conditioning subgroups is thought to be the most appropriate index of place conditioning in the unbiased discrimination procedure used here [26,29,31]. However, because other investigators sometimes use alternative approaches, we conducted two types of additional analyses to assess the presence of CPA within each strain. First, one sample *t*-tests were used to compare each strain’s percent time on the ethanol-paired floor to a hypothetical no-preference value of 50% (“PDT vs. 50%” in Supplementary Table S6). The conclusions yielded by this approach differed from those derived from the comparisons between conditioning subgroups in only one case at each dose (2 g/kg: CBA/J; 4 g/kg: C57BL/6J), i.e., the conclusions were identical for 93% (27/29) of the groups. Second, separate comparisons between the saline-only group and each conditioning subgroup were made using raw grid time scores (“G± vs Saline-Only Group” in Supplementary Table S6). With this approach, the conclusion was identical for 55% (16/29) of the groups. However, the saline-only group differed significantly from one of the subgroups in seven additional cases that showed a significant difference between the conditioning subgroups (24%).

Finally, Supplementary Tables S1, S4, and S5 also list the mean activity rates during the 30-min preference test for the 0-, 2-, and 4-g/kg groups, respectively (collapsed across conditioning subgroups). As on the fourth 5-min conditioning trial (see Figure 2), test activity was inversely related to ethanol dose received during the conditioning phase. Moreover, strain differences in test activity were generally similar to those observed during conditioning. Two-way (Strain × Dose) ANOVA of test activity showed significant main effects of Strain [F(13, 818) = 133.2, *p* < 0.001] and Dose [F(2, 818) = 75.7, *p* < 0.001], as well as a significant Strain × Dose interaction [F(26, 818) = 3.6, *p* < 0.001]. Simple main effect tests at each strain indicated that the dose effect on test activity was significant for the following strains: 129P3/J, AKR/J, BALB/cJ, C58L/J, DBA/1J, DBA/2J, NZB/B1NJ, and SJL/J (all *p*’s < 0.05).

### 3.4. Genetic Correlations

#### 3.4.1. Activity rates

Genetic correlations among the various activity rate phenotypes recorded during the habituation (HAB) and conditioning sessions (C1, C4) for each dose group are shown in Supplementary Table S7. There were significant positive correlations across these phenotypes, both between dose groups and within each dose group. As might be expected, these correlations were highest for phenotypes measured at the same point in training (HAB: All *r*’s = 0.99; C1: *r*’s = 0.98–0.99; C4: 0.89 ≤ *r* ≤ 0.96) than for phenotypes measured at different points in training (e.g., HAB vs. C4: 0.58 ≤ *r* ≤ 0.69). The change in activity rate between HAB and C4 (C4–HAB in Supplementary Table S7) was negatively correlated with HAB and C1 activity rates. That is, strains showing higher activity rates during HAB tended to show larger decreases in activity across conditioning sessions. In contrast, there were no significant correlations between C4 activity rate and C4–HAB (–0.27 ≤ *r* ≤ –0.08).

#### 3.4.2. Conditioned Aversion Test

**Figure 5:**
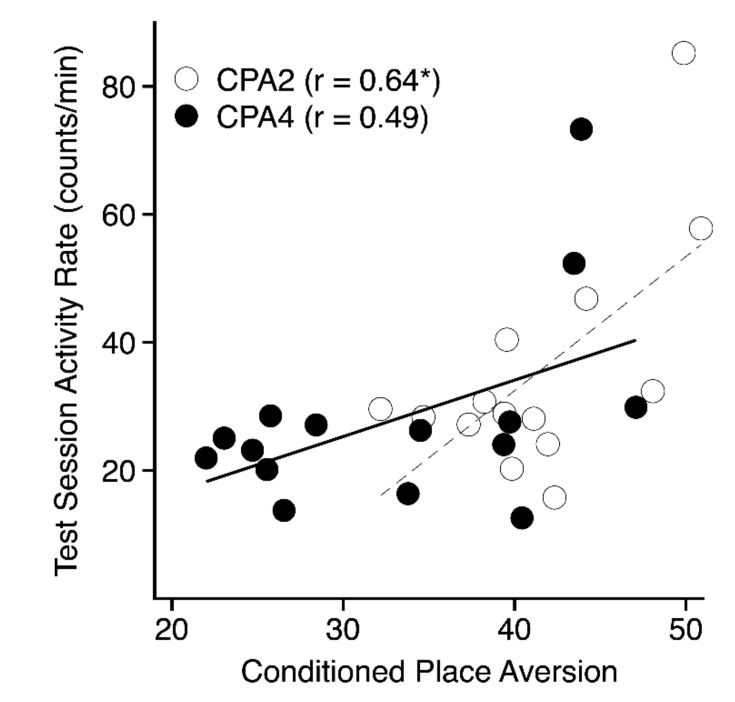
Scatterplot depicting the genetic correlations between mean test session activity rates (y-axis) and conditioned place aversion indexed as mean percent time on the ethanol-paired floor (x-axis) for the 2- (open symbols, dashed line) and 4- (closed symbols, solid line) g/kg groups. Each point represents the strain mean scores for both phenotypes.

Table 1 lists genetic correlations between phenotypes measured during the final preference test and activity rate phenotypes recorded during training and testing. There was no significant correlation (–0.12 ≤ *r* ≤ 0.17) between CPA in either the 2- (PDT-2) or 4-g/kg (PDT-4) groups and test performance in the 0 g/kg group, indexed either as time spent on the grid floor (GT-0) or as percent time on the CS+ floor (PDT-0). However, there was a significant positive correlation between CPA in the 2- and 4-g/kg groups (*r* = 0.67, *p* < 0.01). Analysis also showed several significant genetic correlations between CPA in the 2-g/kg group (PDT-2) and test activity (TACT: 0.54 ≤ *r* ≤ 0.65), as well as with activity on the fourth conditioning trial (C4: 0.56 ≤ *r* ≤ 0.60. In general, stronger aversions (i.e., low PDT values) were associated with lower activity rates (see Figure 5).

#### 3.4.3. Other Ethanol Phenotypes

**Figure 6:**
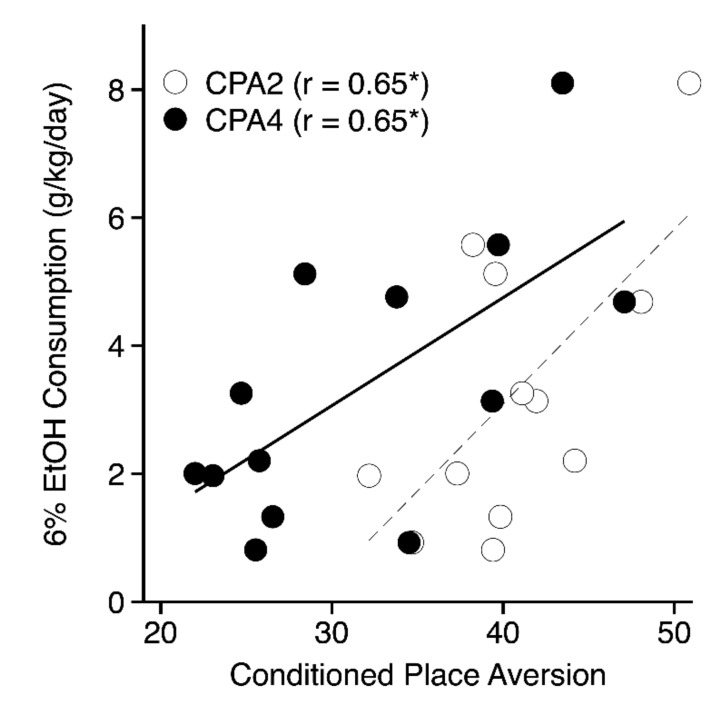
Scatterplot depicting the genetic correlations between mean 6% ethanol consumption [5] (y-axis) and conditioned place aversion indexed as mean percent time on the ethanol-paired floor (x-axis) for the 2- (open symbols, dashed line) and 4- (closed symbols, solid line) g/kg groups. Each point represents the strain mean scores for both phenotypes.

**Figure 7:**
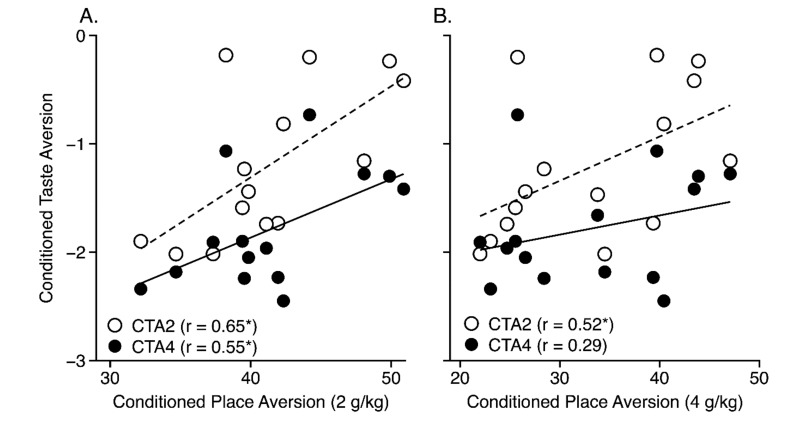
Scatterplots showing the genetic correlations between ethanol-induced conditioned taste aversion indexed as the change in fluid intake between the first and second taste conditioning trial [13] (y axis) and conditioned place aversion indexed as mean percent time on the ethanol-paired floor (x-axis) for the (**A**) 2- and (**B**) 4-g/kg conditioned place aversion groups. The open symbols and dashed lines represent mice given taste conditioning with 2 g/kg, whereas the closed symbols and solid lines represent mice given taste conditioning with 4 g/kg. Each point represents the strain mean scores for both phenotypes.

**Figure 8:**
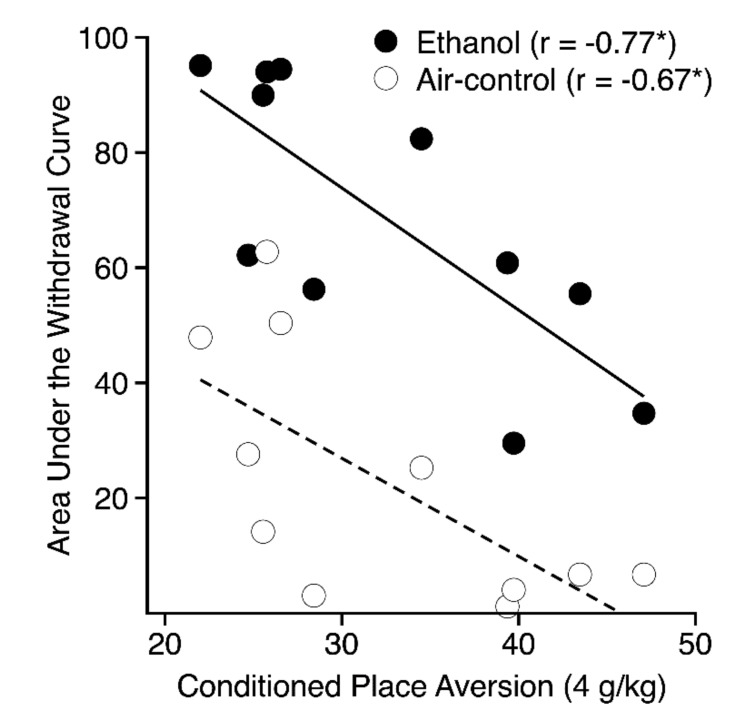
Scatterplot showing the genetic correlations between ethanol withdrawal expressed as uncorrected area under the withdrawal curve [10] (y-axis) and conditioned place aversion indexed as mean percent time on the ethanol-paired floor (x-axis) for the 4-g/kg conditioned place aversion groups. The open symbols and dashed lines represent mice in the air-control groups, whereas the closed symbols and solid lines represent mice in the chronic ethanol withdrawal groups. Each point represents the strain mean scores for both phenotypes.

Table 2 lists genetic correlations between CPA at 2- (PDT-2) or 4- (PDT-4) g/kg and several ethanol-related phenotypes measured in previous multi-strain studies, including ethanol drinking [5], ethanol-induced CTA [13], ethanol-induced CPP [14], blood ethanol concentration (BEC) [2], and drug withdrawal severity [9,10,32]. No significant genetic correlations emerged from the comparisons between CPA and CPP or between CPA and BEC across a range of doses. However, these analyses yielded a significant genetic correlation between intake of 6% ethanol [5] and CPA induced by both ethanol doses (Figure 6: both *r*’s = 0.65, *p* < 0.05). Preference for 6% ethanol also showed a significant genetic correlation with CPA at 4 g/kg (*r* = 0.59, *p* < 0.05) and marginal genetic correlation with CPA at 2 g/kg (*r* = 0.52, *p* = 0.08). In addition, there were significant genetic correlations between ethanol-induced CTA [13] and ethanol-induced CPA (Figure 7: 0.52 ≤ *r* ≤ 0.65, *p* < 0.05). Interestingly, CPA in the 4-g/kg groups was genetically correlated (Figure 8: −0.77 ≤ *r* ≤ −0.67, *p* < 0.05) with seizure sensitivity (as indexed by handling-induced convulsions, HICs) both in ethanol-exposed mice and in air control mice from a study of chronic ethanol withdrawal induced by 72-h exposure to ethanol vapor or air [10]. Although the genetic correlation between CPA at 4 g/kg and acute ethanol withdrawal after a 4-g/kg injection [9] fell short of the criterion for significance (*r* = −0.53, *p* = 0.07), CPA was genetically correlated with precipitated diazepam withdrawal measured in two separate studies (−0.66 ≤ *r* ≤ −0.63, *p* < 0.05) [9,32].

## 4. Discussion

### 4.1. Conditioned Place Aversion

This study is the first to examine mouse strain differences in ethanol-induced CPA. Analysis revealed dose-dependent development of CPA (Figure 3) and a significant main effect of genotype (Figure 4) at both ethanol doses in a large panel of genetically diverse inbred mouse strains (*n* = 15). The strong genetic correlation between CPA in the 2- (PDT-2) and 4- (PDT-4) g/kg groups (Table 1: *r* = 0.67, *p* < 0.01) indicates overlap in the genes underlying the conditioned aversive response to each dose [25]. Within-strain comparisons of the counterbalanced conditioning subgroups confirmed that expression of CPA resulted from an associative learning process in a majority of the strains at each dose (Supplementary Table S6). Moreover, the lack of genetic correlation between test performance in saline-treated control mice (Group 0) and CPA in the ethanol-treated groups suggests that strain differences in ethanol-induced CPA were not secondary to strain differences in unconditioned preferences for the grid and hole floor cues. Despite clear evidence for a genetic influence, heritabilities for CPA (indexed by percent EtOH time) at each dose were relatively low (*h* = 0.06 and 0.14 for the 2- and 4-g/kg doses, respectively), indicating a large contribution of environmental factors to this particular behavioral phenotype.

Induction of CPA by post-trial injection of ethanol in several different inbred mouse strains extends the generalizability of this phenomenon well beyond the single strain (DBA/2J) used in previous studies [19,20,21,22,23,24]. As discussed in those reports, this conditioning effect is perhaps best explained by suggesting that ethanol injection produces a time-dependent bivalent motivational effect that is initially aversive (immediately after injection), but rapidly becomes rewarding as brain ethanol concentration increases. Presumably, mice injected with ethanol immediately after CS exposure form an association between the CS and the initial aversive effect of ethanol injection (resulting in CPA), whereas mice injected a few seconds before CS exposure form an association between the CS and the slightly delayed rewarding effect of ethanol injection (resulting in CPP). Although the mechanisms underlying the initial aversive effect are unknown, we have previously proposed that this effect may be related to the unexpected novelty of the rapid transition from a sober state to an intoxicated state immediately after exposure to the CS. Furthermore, we have argued that this short-lived initial aversive effect of ethanol injection is mechanistically independent of the slightly delayed, longer-lasting rewarding effect. This interpretation is supported by the finding that home cage injections of ethanol during the week before place conditioning impair the later development of CPA (presumably because the transition from sober to drunk is no longer novel), but have no detectable effect on the development of CPP [22]. The conclusion that CPA and CPP are mechanistically unrelated is further supported by the lack of genetic correlation between these phenotypes (Table 2).

### 4.2. Activity Phenotypes

In contrast to CPA, heritabilities for the primary activity phenotypes (Supplementary Tables S1–S3) were much higher (0.41 ≤ h ≤ 0.79), which is generally consistent with the high heritabilities previously reported for similar activity phenotypes in a study of ethanol-induced CPP in the same 15 inbred strains [14]. As might be expected, there were high genetic correlations among the various activity phenotypes (Supplementary Table S7, Table 1). Of particular interest, there were significant genetic correlations between CPA in the 2-g/kg groups (PDT-2) and test activity scores in all three dose groups (Table 1: 0.54 ≤ *r* ≤ 0.65). More specifically, strains that showed stronger CPA (i.e., lower PDT values) were less active during test sessions compared to strains that showed weaker CPA. Those correlations are consistent with two previous multi-strain studies of CPP that also showed significant genetic correlations between place conditioning and test activity [11,14]. In the case of CPP, stronger preferences (i.e., higher PDT values) were associated with lower activity rates. Taken together, these correlations suggest that low levels of test session activity are genetically associated with stronger expression of place conditioning, regardless of whether the learning is based on a rewarding or aversive outcome. That observation is also consistent with previous CPP studies suggesting that environmental manipulations that alter test activity similarly affect expression of place conditioning in rodents (e.g., [33,34]). As noted previously, the simplest explanation for these findings might be that CPA (or CPP) and activity are competing responses, and that test activity is affected by both environmental manipulations and genotype [14].

The large reduction in activity during the CS between the first and last conditioning trials in saline-treated mice (0 g/kg) most likely reflects habituation to the apparatus and handling (Figure 2). However, the greater dose-dependent decrease in activity in ethanol-treated mice is of particular interest because it raises the possibility that, in addition to inducing CPA, post-trial ethanol injection produced a conditioned suppression of activity during the CS. This hypothesized conditioned activity reduction was presumably superimposed on the normal habituation process, resulting in lower levels of activity on C4 in the 2- and 4-g/kg groups than in the saline group. It is worth noting that this experiment was not really designed to address the putative associative basis for this lower activity response since the saline control group was not matched to the ethanol groups for overall exposure to ethanol (e.g., by giving ethanol injections in the home cage), leaving open the possibility that lower activity rates in the ethanol groups were simply a non-associative effect of repeated ethanol exposure. The failure to see any difference between activity rates on CS+ and CS−trials also argues against an associative account, although that lack of difference could be due to a floor effect. However, it should be noted that a previous CPP study showed that the place conditioning apparatus could acquire the ability to elicit a conditioned activity response even when no difference between activity on CS+ and CS−trials could be detected [35].

### 4.3. Genetic Correlations with Other Ethanol Phenotypes

Importantly, analysis showed no genetic correlations between ethanol-induced CPA and BEC across a range of ethanol doses, suggesting that strain differences in CPA were not driven simply by strain differences in ethanol pharmacokinetics. In contrast, analysis revealed several interesting genetic correlations between ethanol-induced CPA and other ethanol-related phenotypes (Table 2). As expected, there were positive genetic correlations between CPA and ethanol-induced CTA [13], and negative genetic correlations between CPA and ethanol drinking and preference [5]. The genetic correlations between CPA and CTA suggest overlap in the genetic mechanisms underlying these aversion-related phenotypes. The finding that both CPA (Table 2) and CTA [13] are negatively correlated with ethanol intake and preference [5] offers additional support for the idea of commonality in their underlying mechanisms. Overall, these data add to the growing body of evidence suggesting that individual differences in ethanol intake and preference are determined, at least in part, by variations in sensitivity to ethanol’s aversive effects [13,17,18].

Given the positive genetic correlation between CPA and CTA and the fact that both procedures involve learning, consideration must be given to the possibility that this relationship is driven by genetic differences in learning, instead of differences in sensitivity to ethanol’s aversive effects. However, several lines of evidence argue against this interpretation. First, analysis revealed no genetic correlation between CPA and ethanol-induced CPP (Table 2) in the same inbred strain panel [14], even though similar Pavlovian learning processes were involved in both procedures. To further address this issue, strain means for ethanol-induced CPA were also correlated with strain means from several learning studies in which at least five of the strains used here were tested (Table 3). As can be seen, there were only three statistically significant (*p* < 0.05) genetic correlations with CPA in 2-g/kg groups (PDT-2) that were in the direction predicted by the learning hypothesis [36,37], but there were no significant correlations with CPA in the 4-g/kg groups (PDT-4). Moreover, there were several other learning procedures that showed no significant genetic correlations with ethanol-induced CPA [37,38,39,40,41]. Although the learning hypothesis cannot be completely dismissed on the basis of these data, the failure to find any significant genetic correlations in the 4-g/kg groups or between CPA and many different learning measures (including CPP) suggests that the genetic correlations between CPA and CTA are not easily explained solely in terms of strain differences in learning ability.

The finding that ethanol-induced CPA in the 4-g/kg groups was genetically correlated with several different measures of acute or chronic drug withdrawal (Table 2) in three different studies [9,10,32] is of interest because previous inbred strain studies have shown significant genetic correlations between ethanol-induced CTA and acute or chronic ethanol withdrawal [12,13,18]. In all of these cases, stronger CPA or CTA was associated with greater withdrawal severity. It must be noted, however, that for most of the genetic correlations with CPA, the withdrawal phenotype was not corrected for baseline strain differences in sensitivity to handling-induced convulsions (HICs). Indeed, the study of chronic ethanol withdrawal yielded a significant genetic correlation between ethanol-induced CPA and HICs in control mice that were not exposed to ethanol, implying commonality in the genes underlying CPA and general seizure susceptibility rather than a more selective sensitivity to seizures elicited during drug withdrawal. The overall pattern of relationships among CPA, CTA, and withdrawal suggests overlap in the neurobiological mechanisms mediating sensitivity to ethanol’s aversive effects and sensitivity to events that elicit seizures.

Interestingly, previous studies have shown that sensitivity to ethanol withdrawal is also genetically correlated with ethanol drinking and with CPP. More specifically, mouse strains or selectively bred mouse lines that show more severe withdrawal drink less ethanol than strains/lines that show less severe withdrawal [42]. In contrast, strains or lines that show more severe withdrawal display stronger CPP than strains or lines showing less severe withdrawal [14,43,44]. Although these genetic correlations seem to be at odds with one another, one possible interpretation is that ethanol drinking and CPP are correlated with different subsets of genes influencing withdrawal sensitivity. That is, ethanol drinking may reflect the influence of genes that underlie sensitivity to ethanol’s aversive effects (i.e., CPA and CTA) and one subset of genes controlling ethanol withdrawal, whereas CPP reflects the influence of genes that underlie sensitivity to ethanol’s rewarding effects and a different subset of genes controlling ethanol withdrawal. Clearly, more research is needed to fully understand the genetic relationships among these ethanol-induced behavioral phenotypes.

## 5. Conclusions

In summary, the present study strongly supports the hypothesis of commonality in the neurobiological mechanisms that mediate ethanol-induced CPA and CTA. Furthermore, these data offer convergent support for the idea that genetic differences in sensitivity to ethanol’s aversive effects contribute to the role that genotype plays in determining ethanol intake and preference in mice. Strains that are more sensitive to ethanol’s aversive effects, whether indexed by CPA or CTA, voluntarily drink less ethanol and show weaker preference for ethanol compared to strains that are less sensitive to ethanol’s aversive effects. Finally, the associations among these ethanol-induced aversive effects, ethanol drinking/preference, and seizure susceptibility suggest that future research on this nexus of phenotypes may increase understanding of the neurobiological mechanisms that protect against development of alcohol use disorders and possibly aid in the identification of new approaches for treating such disorders.

## Figures and Tables

**Table 1:** Genetic correlations (Pearson *r*) between conditioned place aversion (CPA) and activity phenotypes.

Phenotype *	GT-0	PDT-0	PDT-2	PDT-4	TACT-0	TACT-2	TACT-4
**GT-0**	--	--	--	--	--	--	--
**PDT-0**	0.14	--	--	--	--	--	--
**PDT-2**	0.11	0.09	--	--	--	--	--
**PDT-4**	−0.12	0.17	**0.67^b^**	--	--	--	--
**TACT-0**	0.35	0.44	**0.54^a^**	0.25	--	--	--
**TACT-2**	0.38	0.40	**0.64^a^**	0.35	**0.96^c^**	--	--
**TACT-4**	0.34	0.42	**0.65^a^**	0.49	**0.88^c^**	**0.96^c^**	--
**HAB-0**	0.14	0.33	0.35	0.32	**0.76^b^**	**0.82^c^**	**0.81^c^**
**HAB-2**	0.21	0.29	0.37	0.30	**0.75^b^**	**0.83^c^**	**0.83^c^**
**HAB-4**	0.13	0.32	0.35	0.30	**0.71^b^**	**0.79^c^**	**0.78^c^**
**C4-0**	0.44	0.44	**0.56^a^**	0.14	**0.86^c^**	**0.82^c^**	**0.71^b^**
**C4-2**	0.39	0.41	**0.59^a^**	0.18	**0.73^b^**	**0.78^c^**	**0.69^b^**
**C4-4**	0.43	0.49	**0.60^a^**	0.26	**0.79^c^**	**0.85^c^**	**0.81^c^**
**(C4-HAB)-0**	0.11	−0.12	−0.07	−0.30	−0.37	−0.47	**−0.54^a^**
**(C4-HAB)-2**	−0.02	−0.11	−0.11	−0.26	−0.50	**−0.57^a^**	**−0.62^a^**
**(C4-HAB)-4**	0.09	−0.12	−0.11	−0.23	−0.45	**−0.54^a^**	**−0.52^a^**
c = *p* < 0.001, b = *p* < 0.01, a = *p* < 0.05; significant correlations are shown in **boldface**							

* The suffix (-0, -2, -4) indicates the dose group; GT-0 = test time on grid floor in saline group; PDT = percent time on ethanol-paired floor; TACT = test session activity rate. See Supplementary Table S7 for definitions of other activity phenotypes.

**Table 2:** Genetic correlations (Pearson *r*) between CPA and other ethanol phenotypes.

Phenotype	*n*	CPA PDT-2	CPA PDT-4
Belknap et al. (1993)			
3% ethanol intake	12/13	0.27	0.52 *
6% ethanol intake	12/13	**0.65^a^**	**0.65^a^**
10% ethanol intake	12/13	0.41	0.30
3% ethanol preference ratio	12/13	0.08	0.44
6% ethanol preference ratio	12/13	0.52 *	**0.59^a^**
10% ethanol preference ratio	12/13	0.39	0.32
Broadbent et al. (2002)			
Conditioned Taste Aversion (2 g/kg)	14/15	**0.65^a^**	**0.52^a^**
Conditioned Taste Aversion (4 g/kg)	14/15	**0.55^a^**	0.29
Crabbe et al. (1994)			
Blood Ethanol Concentration (1 g/kg)	12/13	0.19	0.32
Blood Ethanol Concentration (2 g/kg)	12/13	−0.25	−0.15
Blood Ethanol Concentration (3 g/kg)	12/13	0.18	0.45
Blood Ethanol Concentration (4 g/kg)	12/13	0.31	0.42
Cunningham (2014)			
Conditioned Place Preference (2 g/kg)	14/15	−0.27	−0.09
Conditioned Place Preference (4 g/kg)	14/15	−0.17	−0.09
Metten & Crabbe (1994)			
Acute Ethanol Withdrawal AREA (uncorrected)	12/13	−0.03	−0.53 *
Acute Ethanol Withdrawal Peak (uncorrected)	12/13	0.02	−0.48 *
Acute Diazepam Withdrawal Peak (uncorrected)	12/13	−0.29	**−0.66^a^**
Metten & Crabbe (1999)			
Acute Diazepam Withdrawal Peak (uncorrected)	12/13	−0.30	**−0.63^a^**
Metten & Crabbe (2005)			
Air Control AREA 25	11	−0.20	**−0.67^a^**
Chronic Ethanol Withdrawal AREA 25 (uncorrected)	11	−0.37	**−0.77^a^**
Chronic Ethanol Withdrawal (Δ AREA25)	11	−0.29	−0.24

**Boldface** indicates a significant correlation (a = *p* < 0.05). * 0.05 < *p* < 0.10.

**Table 3:** Genetic correlations (Pearson *r*) between CPA and learning phenotypes.

Reference	Phenotype	*n*	CPA PDT-2 *	CPA PDT-4 *
Wong & Brown (2006)				
	Visual detection task (D8 % correct)	6	−0.69	−0.05
	Visual detection task (D8 latency)	6	**0.86^a^**	−0.22
	Pattern discrimination task (D8 % correct)	6	−0.62	−0.16
	Pattern discrimination task (D8 latency)	6	**0.92^a^**	−0.22
	Swim speed (cm/sec)	6	−0.55	−0.10
Brown & Wong (2007)				
	Visual detection task (D8 % correct)	5	−0.58	−0.29
	Pattern discrimination task (D8 % correct)	5	−0.46	−0.41
	Reversal latency in MWM (sec)	5	0.81	0.09
	Reversal swim distance in MWM (cm)	5	**0.92^a^**	0.10
	Swim speed (cm/sec)	5	−0.17	−0.44
	Probe trial in MWM (% time in correct quadrant)	5	−0.77	−0.02
	Probe trial in MWM (annulus crossings)	5	−0.85	0.31
	Latency to visible platform in MWM (sec)	5	0.79	0.04
	Conditioned odor preference task (% CS+ digging)	5	0.10	0.68
	Latency to fall on the Rotarod-males (sec)	5	−0.28	0.26
O’Leary et al. (2011)				
	Acquisition latency (s)	6	0.28	−0.46
	Reversal latency (s)	6	−0.01	−0.10
	Reversal difference latency	6	−0.71	0.41
	Number of acquisition errors	6	−0.02	−0.37
	Number of reversal errors	6	0.06	−0.06
	Reversal difference errors	6	−0.81	0.39
	% time correct quadrant	6	−0.67	0.52
Portugal et al. (2011)				
	Contextual Fear Conditioning (% immobility)	5	0.23	−0.06
	Cued Fear Conditioning (% immobility)	5	0.66	0.33
Pinhas et al. (2012)				
	CS+Sucrose (% CS+ intake)	7	−0.16	−0.63
	CS+Fructose (% CS+ intake)	7	0.23	−0.09
	CS+Saccharin (% CS+ intake)	7	0.11	−0.31
Ishiwatari & Bachmanov (2012)				
	CS intake (mL) Difference (Trial 1–Trial 2)	6	0.66	0.41

* Percent time on ethanol-paired floor for 2- (PDT-2) or 4- (PDT-4) g/kg groups. **Boldface** indicates a significant correlation (a = *p* < 0.05).

## Supplementary Materials

The following are available online at https://www.mdpi.com/2076-3425/9/8/209/s1, Table S1: Saline (0 g/kg) group strain means (± SEM) for activity (counts/min) during habituation, conditioning and testing and for time spent on the grid floor (s/min) during testing; Table S2: 2 g/kg group strain means (± SEM) for activity (counts/min) during habituation and conditioning; Table S3: 4 g/kg group strain means (± SEM) for activity (counts/min) during habituation and conditioning; Table S4: 2 g/kg group strain means (± SEM) for time spent on the grid floor (s/min), percent time on the ethanol floor and activity (counts/min) during testing; Table S5: 4 g/kg group strain means (± SEM) for time spent on the grid floor (s/min), percent time on the ethanol floor and activity (counts/min) during testing; Table S6: Summary of analyses to determine whether significant CPA was obtained in each strain (see main text); Table S7: Genetic correlations (Pearson r) between activity phenotypes during habituation and conditioning.

## Appendix A

The number of A/HeJ mice was lower than in the other strains tested here due to reduced availability from the Jackson Laboratory during this study. In addition, after completing one of the cohorts that included A/HeJ mice, Jackson Laboratory reported that one of the male breeders used to generate our A/HeJ mice may have been tainted with genetic material from a different strain, requiring that we remove data from all A/HeJ mice tested in that cohort. Due to the reduced availability of this strain, mice were assigned only to the 0 and 4 g/kg groups.
