# Supplementary material for: Genetic Relationships Between Ethanol-Induced Conditioned Place Aversion and Other Ethanol Phenotypes in 15 Inbred Mouse Strains
Source: Brain Sci. 2019 Aug 20;9(8):209. doi: 10.3390/brainsci9080209 (PMC6721285; doi:10.3390/brainsci9080209)
Supplement: Supplementary file 1 [file brainsci-09-00209-s001.pdf]

Table S1: Saline (0 g/kg) group strain means (± SEM) for activity (counts/min) during habituation, conditioning and testing and for time spent on the grid floor (s/min) during testing. .

Strain	*n*#	Habituation (activity/min)	Saline: Trial 1* (activity/min)	Saline: Trial 4* (activity/min)	Preference Test (s/min)	Test Activity (activity/min)
129P3/J	12	75.0 ± 4.8	54.9 ± 2.9	38 ± 3.7	29.6 ± 3.2	32.2 ± 2.7
A/HeJ	9	57.6 ± 5.1	47.2 ± 3.5	29.5 ± 2.6	31.4 ± 2.9	32.0 ± 2.7
AKR/J	12/13	97.1 ± 5.1	74.1 ± 4.4	46.9 ± 5.9	27.8 ± 1.4	50.3 ± 2.3
BALB/cJ	13	83.6 ± 4.0	70.8 ± 2.2	65.4 ± 2.7	33.4 ± 1.6	63.9 ± 2.7
C3H/HeJ	13	35.8 ± 1.7	33.2 ± 1.8	25.1 ± 2.9	31.2 ± 1.0	28.3 ± 1.2
C57BL/6J	12	112.7 ± 5.9	57.8 ± 5.1	23.7 ± 2.3	28.9 ± 3.5	32.7 ± 2.4
C57L/J	12	117.0 ± 5.0	88.2 ± 4.8	47.0 ± 5.4	28.9 ± 2.4	52.1 ± 4.4
C58/J	12	135.4 ± 10.7	97.8 ± 6.1	66.2 ± 9.0	34.0 ± 2.3	97.9 ± 6.0
CBA/J	13	61.9 ± 5.0	48.7 ± 1.3	34.3 ± 3.1	32.8 ± 1.7	30.9 ± 1.3
DBA/1J	12	80.9 ± 6.9	67.1 ± 6.1	37.7 ± 7.0	30.6 ± 2.7	42.1 ± 3.1
DBA/2J	12	54.5 ± 5.3	50.1 ± 3.8	35.4 ± 5.3	26.7 ± 3.0	37.0 ± 3.4
NZB/B1NJ	12	54.9 ± 3.5	39.7 ± 3.1	18.7 ± 3.9	19.7 ± 2.4	22.6 ± 2.1
PL/J	13	66.0 ± 3.6	64.0 ± 3.8	50.7 ± 5.4	33.0 ± 1.6	34.2 ± 2.4
SJL/J	11/12	51.2 ± 4.6	37.7 ± 2.7	20.5 ± 1.4	32.8 ± 2.8	24.7 ± 1.2
SWR/J	12	76.2 ± 3.9	41.6 ± 2.4	30.5 ± 2.0	32.2 ± 2.0	30.8 ± 1.2
Grand Mean		77.4 ± 2.4	58.4 ± 1.7	38.3 ± 1.6	30.2 ± 0.6	40.9 ± 1.6
Heritability		0.70	0.67	0.46	0.17	0.79

*These scores are the mean activity on the first two saline trials (“Trial 1”) or the last two saline trials (“Trial 4”)

#The second number (if listed) indicates n during preference testing; the first number (conditioning trials) is lower due to equipment errors.

Table S2: 2 g/kg group strain means (± SEM) for activity (counts/min) during habituation and conditioning.

Strain	*n**	Habituation	Saline: Trial 1	Saline: Trial 4	Ethanol: Trial 1	Ethanol: Trial 4
		(activity/min)	(activity/min)	(activity/min)	(activity/min)	(activity/min)
129P3/J	25	78.2 ± 3.7	50.1 ± 3.7	23.2 ± 2.1	52.6 ± 2.9	29.9 ± 2.6
A/HeJ	-	-	-	-	-	-
AKR/J	24	87.8 ± 3.8	70.0 ± 3.1	40.3 ± 2.9	64.2 ± 3.2	42.1 ± 3.2
BALB/cJ	24	81.5 ± 2.9	61.8 ± 2.8	46.4 ± 3.1	67.7 ± 2.3	48.7 ± 2.9
C3H/HeJ	25	30.2 ± 1.5	32.3 ± 2.3	19.7 ± 1.8	33.8 ± 2.1	17.8 ± 1.8
C57BL/6J	24	105.2 ± 4.3	56.5 ± 5.4	17.0 ± 2.3	54.6 ± 4.8	15.8 ± 1.7
C57L/J	25	118.7 ± 3.0	89.2 ± 3.5	50.5 ± 3.4	91.8 ± 5.1	51.0 ± 3.7
C58/J	23	133.6 ± 4.7	93.9 ± 4.2	46.6 ± 5.8	99.1 ± 5.2	48.0 ± 6.6
CBA/J	25	59.6 ± 2.6	49.9 ± 2.6	18.8 ± 2.1	54.8 ± 3.3	19.3 ± 2.0
DBA/1J	26	74.6 ± 4.6	66.8 ± 5.5	20.6 ± 1.8	65.5 ± 3.7	19.1 ± 2.4
DBA/2J	24	53.3 ± 2.9	46.8 ± 3.3	30.6 ± 3.4	48.2 ± 1.6	29.2 ± 3.4
NZB/B1NJ	24	49.5 ± 2.3	36.6 ± 3.3	8.9 ± 1.8	37.0 ± 2.7	9.6 ± 1.9
PL/J	25	66.9 ± 2.5	62.2 ± 3.4	43.7 ± 2.0	62.2 ± 2.0	43.7 ± 1.9
SJL/J	24	48.8 ± 2.9	30.7 ± 2.3	15.7 ± 1.6	34.5 ± 2.1	15.8 ± 1.5
SWR/J	25	78.8 ± 3.2	46.3 ± 2.9	30.4 ± 1.9	48.1 ± 3.2	32.2 ± 2.2
Grand Mean		75.9 ± 1.7	56.6 ± 1.4	29.4 ± 1.0	58.1 ± 1.3	30.1 ± 1.1
Heritability		0.74	0.52	0.50	.57	0.48

*G+ and G- groups combined

Table S3: 4 g/kg group strain means (± SEM) for activity (counts/min) during habituation and conditioning.

**Strain**	***n****	**Habituation**	**Saline:Trial 1**	**Saline: Trial 4**	**Ethanol: Trial 1**	**Ethanol: Trial 4**
		(activity/min)	(activity/min)	(activity/min)	(activity/min)	(activity/min)
129P3/J	25	73.8 ± 4.2	47.1 ± 4	22.7 ± 1.9	48.2 ± 3.5	25.1 ± 2.0
A/HeJ	18	61.6 ± 3.8	45.4 ± 2.9	12.3 ± 2.5	44.8 ± 3.7	8.8 ± 2.3
AKR/J	23	94.1 ± 4.6	63.8 ± 4.0	31.4 ± 3.1	72.9 ± 5.2	32.2 ± 2.5
BALB/cJ	24	82.7 ± 3.1	64.4 ± 3.2	36.2 ± 3.2	70.6 ± 2.6	37.1 ± 3.2
C3H/HeJ	25	32.9 ± 1.6	29 ± 1.9	18.9 ± 1.8	32.4 ± 1.5	17.5 ± 2.2
C57BL/6J	24	106.2 ± 5.6	54.7 ± 4.0	13.6 ± 1.7	57.5 ± 5.7	17.0 ± 1.8
C57L/J	24	114.8 ± 3.7	93.9 ± 5.7	39.7 ± 3.5	92.7 ± 4.8	39.8 ± 3.4
C58/J	24	121.4 ± 5.1	98.6 ± 4.5	47.3 ± 6.6	103.8 ± 3.1	45.8 ± 5.2
CBA/J	25	56.5 ± 2.9	45.4 ± 2.3	16.8 ± 2.0	50.1 ± 2.4	21.6 ± 3.0
DBA/1J	25	73.0 ± 4.6	61.6 ± 5.3	23.1 ± 1.9	71.3 ± 4.1	22.4 ± 2.3
DBA/2J	24	55.0 ± 4.1	46.2 ± 2.8	20.1 ± 2.7	53.7 ± 2.6	22.3 ± 2.4
NZB/B1NJ	24	55.3 ± 2.1	38.8 ± 4.2	8.4 ± 1.4	43.3 ± 3.2	8.8 ± 1.9
PL/J	24	69.7 ± 2.4	61.4 ± 2.5	39.1 ± 2.1	62.0 ± 3.1	38.3 ± 3.2
SJL/J	24	50.2 ± 1.8	30.2 ± 2.6	15.1 ± 1.3	29.7 ± 2.3	11.5 ± 1.4
SWR/J	23	80.9 ± 3.1	44.3 ± 2.2	24.3 ± 1.7	42.5 ± 1.9	29.8 ± 2.7
Grand Mean		75.2 ± 1.6	55.1 ± 1.4	24.8 ± 0.9	58.5 ± 1.4	25.4 ± 0.9
Heritability		0.66	0.55	0.41	0.60	0.41

*G+ and G- groups combined

Table 4: 2 g/kg group strain means (± SEM) for time spent on the grid floor (s/min), percent time on the ethanol floor and activity (counts/min) during testing.

Strain	(n)	G+ (s/min)	(n)	G- (s/min)	Percent EtOH Time*	Test Activity** (activity/min)
129P3/J	13	28.5 ± 4.3	12	44.2 ± 3.4[c]	37.3 ± 5	27.2 ± 1.8
A/HeJ	-	-	-	-	-	-
AKR/J	13	25.6 ± 3.2	13	38.2 ± 3.4[b]	39.5 ± 3.9	40.4 ± 3.0
BALB/cJ	12	31.2 ± 2.9	12	38.1 ± 2.3	44.2 ± 3.4	46.8 ± 2.4
C3H/HeJ	13	31.3 ± 1.7	12	41.5 ± 2.5[a]	41.9 ± 3.3	24.1 ± 1.2
C57BL/6J	12	25.4 ± 3.3	12	39.6 ± 2.5[b]	38.2 ± 3.5	30.7 ± 2.0
C57L/J	12	28.9 ± 2.9	13	28.0 ± 1.8	50.9 ± 2.8	57.8 ± 2.9
C58/J	12	33.1 ± 2.7	11	33.6 ± 2.8	49.9 ± 3.3	85.2 ± 3.3
CBA/J	13	29.7 ± 3.1	12	40.8 ± 3.9[a]	41.1 ± 4.4	28.1 ± 1.4
DBA/1J	14	29.4 ± 3.6	12	49.2 ± 2.5[c]	34.7 ± 4.8	28.3 ± 1.9
DBA/2J	12	24.5 ± 4.1	12	37.2 ± 5.2[b]	39.4 ± 5.4	28.9 ± 1.3
NZB/B1NJ	12	20.6 ± 3.0	12	29.8 ± 4.7	42.3 ± 4.9	15.8 ± 1.3
PL/J	13	28.0 ± 2.3	12	30.3 ± 2.2	48.1 ± 2.6	32.4 ± 1.4
SJL/J	12	18.4 ± 2.8	14	31.4 ± 4.7[b]	39.8 ± 5.0	20.3 ± 1.4
SWR/J	13	22.3 ± 2.2	12	43.9 ± 3.8[c]	32.2 ± 3.6	29.6 ± 2.0
Grand Mean		27.0 ± 0.9		37.4 ± 1.0	41.3 ± 1.1	35.1
Heritability		0.13		0.21	0.06	0.74

*Percent time on ethanol-paired floor for G+ and G- groups combined

**Activity combined for G+ and G- groups

c = $p < .001$, b = $p < .01$, a = $p < .05$, significant difference from G+

Table S5: 4 g/kg group strain means (± SEM) for time spent on the grid floor (s/min), percent time on the ethanol floor and activity (counts/min) during testing.

Strain	(n)	G+ (s/min)	(n)	G- (s/min)	Percent EtOH Time*	Test Activity** (activity/min)
129P3/J	13	14.5 ± 2.7	12	48.2 ± 2.4[c]	22.0 ± 3.0	21.9 ± 1.8
A/HeJ	9	23.2 ± 5.7	9	42.7 ± 4.8[b]	33.8 ± 6.2	16.4 ± 1.7
AKR/J	12	20.1 ± 3.8	12	46.0 ± 4.5[c]	28.4 ± 4.9	27.1 ± 3.2
BALB/cJ	12	17.9 ± 5.0	12	47.0 ± 3.0[c]	25.8 ± 4.8	28.5 ± 3.1
C3H/HeJ	12	24.6 ± 2.0	13	37.3 ± 3.0[a]	39.4 ± 3.0	24.0 ± 1.7
C57BL/6J	12	22.2 ± 3.2	12	34.6 ± 3.9[a]	39.7 ± 4.1	27.6 ± 2.3
C57L/J	12	26.8 ± 2.8	12	34.7 ± 3.3	43.5 ± 3.5	52.3 ± 2.9
C58/J	12	26.8 ± 3.4	12	34.2 ± 4.1	43.9 ± 4.3	73.3 ± 5.6
CBA/J	12	14.5 ± 2.8	13	44.9 ± 3.2[c]	24.7 ± 3.5	23.2 ± 1.5
DBA/1J	12	23.0 ± 2.7	13	41.4 ± 3.9[c]	34.5 ± 4.0	26.3 ± 1.6
DBA/2J	12	14.6 ± 3.7	12	43.9 ± 4.6[c]	25.5 ± 4.8	20.1 ± 1.8
NZB/B1NJ	12	20.1 ± 4.2	12	31.6 ± 5.6[a]	40.4 ± 5.9	12.6 ± 1.1
PL/J	11	27.2 ± 2.2	13	30.8 ± 4.0	47.1 ± 3.9	29.9 ± 1.7
SJL/J	13	11.1 ± 1.7	12	38.8 ± 5.1[c]	26.5 ± 4.6	13.8 ± 1.2
SWR/J	11	15.0 ± 2.9	12	47.2 ± 3.1[c]	23.1 ± 3.5	25.1 ± 2.4
Grand Mean		20.0 ± 0.9		40.1 ± 1.1	33.2 ± 1.2	28.3 ± 1.0
Heritability		0.18		0.16	0.14	0.61

*Percent time on ethanol-paired floor for G+ and G- groups combined

**Activity combined for G+ and G- groups

c = $p < .001$, b = $p < .01$, a = $p < .05$, significant difference from G+

Table S6: Summary of analyses to determine whether significant CPA was obtained in each strain (see main text).

Strain	G+ vs. G-		PDT vs 50%			G± vs. Saline-Only Group			
	2 g/kg	4 g/kg	0 g/kg	2 g/kg	4 g/kg	2:G+	2:G-	4:G+	4:G-
129P3/J	c	c	ns	a	c	ns	a	c	c
A/HeJ	-	a	ns	-	a	-	-	ns	ns
AKR/J	a	c	ns	a	c	ns	a	ns	b
BALB/cJ	ns	c	ns	ns	c	ns	ns	b	a
C3H/HeJ	c	c	ns	a	b	ns	c	ns	ns
C57BL/6J	b	ns	ns	b	a	ns	ns	ns	ns
C57L/J	ns	ns	ns	ns	ns	ns	ns	ns	ns
C58/J	ns	ns	ns	ns	ns	ns	ns	ns	ns
CBA/J	a	c	ns	ns	c	ns	ns	c	b
DBA/1J	c	c	ns	b	c	ns	c	ns	ns
DBA/2J	ns	c	ns	ns	c	ns	ns	ns	b
NZB/B1NJ	ns	ns	ns	ns	ns	ns	ns	ns	ns
PL/J	ns	ns	ns	ns	ns	ns	ns	ns	ns
SJL/J	ns	c	ns	ns	c	a	ns	c	ns
SWR/J	c	c	ns	c	c	a	a	c	c

c = $p < .001$, b = $p < .01$, a = $p < .05$, ns = not significant;

Note: In all cases, the difference showed place aversion, not preference.

Table S7: Genetic correlations (Pearson r) between activity phenotypes during habituation and conditioning.

**Phenotype***	**HAB-0**	**HAB-2**	**HAB-4**	**C1-0**	**C1-2**	**C1-4**	**C4-0**	**C4-2**	**C4-4**	**(C4-HAB)-0**	**(C4-HAB)-2**	**(C4-HAB)-4**
**HAB-0**	--	--	--	--	--	--	--	--	--	--	--	--
**HAB-2**	**0.99**[c]	--	--	--	--	--	--	--	--	--	--	--
**HAB-4**	**0.99**[c]	**0.99**[c]	--	--	--	--	--	--	--	--	--	--
**C1-0**	**0.87**[c]	**0.87**[c]	**0.86**[c]	--	--	--	--	--	--	--	--	--
**C1-2**	**0.88**[c]	**0.89**[c]	**0.86**[c]	**0.98**[c]	--	--	--	--	--	--	--	--
**C1-4**	**0.88**[c]	**0.88**[c]	**0.86**[c]	**0.99**[c]	**0.99**[c]	--	--	--	--	--	--	--
**C4-0**	**0.59**[a]	**0.61**[a]	**0.59**[a]	**0.84**[c]	**0.81**[c]	**0.81**[c]	--	--	--	--	--	--
**C4-2**	**0.58**[a]	**0.63**[a]	**0.63**[a]	**0.79**[c]	**0.78**[c]	**0.77**[c]	**0.89**[c]	--	--	--	--	--
**C4-4**	**0.66**[b]	**0.69**[b]	**0.67**[b]	**0.84**[c]	**0.85**[c]	**0.83**[c]	**0.91**[c]	**0.96c**	--	--	--	--
**(C4-HAB)-0**	**-0.85**[c]	**-0.82**[c]	**-0.83**[c]	**-0.53**[a]	**-0.55**[a]	**-0.56**[a]	-0.08	-0.12	-0.21	--	--	--
**(C4-HAB)-2**	**-0.89**[c]	**-0.88**[c]	**-0.86**[c]	**-0.61**[a]	**-0.64**[a]	**-0.63**[a]	-0.22	-0.17	-0.27	**-0.95**[c]	--	--
**(C4-HAB)-4**	**-0.89**[c]	**-0.89**[c]	**-0.90**[c]	**-0.61**[a]	**-0.63**[a]	**-0.63**[a]	-0.22	-0.27	-0.27	**-0.95**[c]	**-0.95**[c]	--

c = $p < 0.001$, b = $p < 0.01$, a = $p < 0.05$; significant correlations are shown in boldface

*The suffix (-0, -2, -4) indicates the ethanol dose group; HAB = habituation day activity rate; C1 = conditioning trial 1 activity rate; C4 = conditioning trial 4 activity rate; C4-HAB = difference in activity rates between conditioning trial 4 and habituation trial 1

Note: C1 and C4 scores are based on the average of the CS+ and CS- trials (see text for explanation).
